# Acute Q Fever and Scrub Typhus, Southern Taiwan

**DOI:** 10.3201/eid1510.090007

**Published:** 2009-10

**Authors:** Chung-Hsu Lai, Yen-Hsu Chen, Jiun-Nong Lin, Lin-Li Chang, Wei-Fang Chen, Hsi-Hsun Lin

**Affiliations:** E-Da Hospital and I-Shou University, Kaohsiung City, Taiwan (C.-H. Lai, J-.N. Lin, W.-F. Chen, H.-H. Lin); Kaohsiung Medical University, Kaohsiung City (C.-H. Lai, Y-H. Chen, J.-N. Lin, L.-L. Chang); National Yang-Ming University, Taipei, Taiwan (H.-H. Lin)

**Keywords:** Q fever, scrub typhus, Coxiella burnetii, Orientia tsutsugamushi, co-infection, Taiwan, vector-borne infections, bacteria, dispatch

## Abstract

Acute Q fever and scrub typhus are zoonoses endemic to southern Taiwan. Among the 137 patients with acute Q fever (89, 65.0%) or scrub typhus (43, 31.4%), we identified 5 patients (3.6%) who were co-infected with *Coxiella burnetii* and *Orientia tsutsugamushi*.

Q fever is a worldwide zoonosis in humans caused by *Coxiella burnetii* infection. Ticks are the main arthropod vectors of *C. burnetii*; the major animal reservoirs include goats, sheep, cattle, and domestic cats. Humans are infected mainly by inhaling organism-contaminated aerosols (*1*). Scrub typhus, caused by *Orientia tsutsugamushi* infection, is endemic to eastern Asia and the western Pacific region. *O. tsutsugamushi* is transmitted vertically in mites (particularly *Leptotrombidium* species) by the transovarial route, and horizontally in rodents through trombiculid larval (chigger) bites. Humans contract scrub typhus by being bitten by chiggers infected with *O. tsutsugamushi;* such bites occur accidentally during agriculture or field recreational activities (*2*).

Although the major arthropod vectors, animal reservoirs, and routes of transmission to humans are different for *C. burnetii* and *O. tsutsugamushi*, co-infection may occur when humans have been exposed to an environment where arthropod vectors and animal reservoirs are prevalent. In southern Taiwan, acute Q fever and scrub typhus are endemic zoonoses (*3**–**5*), and co-infection with the 2 pathogens may occur. We report 5 cases of co-infection with the agents of acute Q fever and scrub typhus.

## The Study

This study was conducted at E-Da Hospital, a teaching hospital located in Kaohsiung County in southern Taiwan, and approved by its Institute Ethics Committee (E-MRP-096-065) in 2006. Rickettsial diseases are notifiable diseases in Taiwan, and suspected cases with appropriate clinical characteristics are reported to the Center for Disease Control, Taipei, Taiwan for confirmation. Because acute Q fever, scrub typhus, and murine typhus (caused by flea-borne *Rickettsia typhi*) are the most common rickettsial diseases in Taiwan (*3*–*5*), we requested tests for the etiologic agents of the 3 diseases simultaneously in patients who sought treatment, regardless of which disease we suspected on the basis of clinical features.

Serologic assessments for specific antibodies to *C*. *burnetii* and *O*. *tsutsugamushi* were performed by using indirect immunofluorescence antibody assay as previously described (*5*). Acute Q fever was diagnosed by either an antiphase II antigen immunoglobulin (Ig) G titer >320 and antiphase II antigen IgM titer >80 in a single serum sample, or a >4-fold rise of antiphase II antigen IgG titer in paired serum samples. Antigens of 3 major strains of *O. tsutsugamushi* (Karp, Kato, and Gilliam strains) were used to diagnose scrub typhus: either an IgM titer >80 or a >4-fold rise in IgG titer in paired serum samples for Karp, Kato, and Gilliam strains of *O. tsutsugamushi*. Murine typhus was diagnosed by an IgM titer >80 or a >4-fold rise in IgG titer against *R. typhi* in paired sera. Co-infection with *C. burnetii* and *O. tsutsugamushi* was diagnosed if the serologic results fulfilled the diagnostic criteria for both infections.

From April 15, 2004, through June 30, 2008, we identified 12 cases of murine typhus, 89 cases of acute Q fever, and 43 cases of scrub typhus; 5 persons had both acute Q fever and scrub typhus. All 5 patients with co-infections denied having fever within 3 months before admission. The demographic data and clinical manifestations of the 5 case-patients co-infected with *C. burnetii* and *O. tsutsugamushi* are listed in the Appendix Table). Thrombocytopenia and elevated liver enzyme levels improved after antimicrobial drug treatment.

## Conclusions

In this study, we found that 5.3% (5/94) and 10.4% (5/48) patients who fulfilled the serologic diagnosis of acute Q fever and scrub typhus, respectively, were co-infected with *C. burnetii* and *O. tsutsugamushi*. Although the major arthropod vectors, animal reservoirs, and route of transmission to humans are different for *C. burnetii* and *O. tsutsugamushi*, co-infection with these 2 organisms can occur, particularly in regions where both Q fever and scrub typhus are endemic, such as southern Taiwan. Identification of co-infection improves understanding of the epidemiology of each disease and reminds clinicians to monitor patients for whom the development of chronic Q fever after acute Q fever is a high risk; such follow-up is not needed for the cases of scrub typhus alone.

Few cases of concurrent Q fever and other rickettsioses have been reported in the literature (6*,**7*). Rolain et al. found that 6 patients with tick-borne rickettsioses had concomitant or consecutive infection with *C. burnetii*; antibodies were routinely tested against antigens of *C. burnetii, Rickettsia* spp.*, Anaplasma phagocytophylum, Francisella tularensis,* and *Borrelia burgdorferi* (*6*). Five and 2 of their 6 patients had an eschar and skin rash, respectively, and none had a history or clinical signs suggestive of acute Q fever. In contrast, none of our patients had an eschar, the characteristic manifestation of scrub typhus, and only 1 had a skin rash, which made a presumptive clinical diagnosis of scrub typhus difficult.

For scrub typhus, co-infection with *Leptospira* spp. (*8**–**12*) and *R. typhi* (*13*) has been reported. Co-infection with *O. tsutsugamushi* and *Leptospira* spp. tends to be associated with severe illness and death (*8**–**12*), and cases are mainly reported in Southeast Asia, particularly in Thailand (*8*) and Taiwan (*9**–**12*). However, we did not routinely test for leptospirosis in this study. Cases of *O. tsutsugamushi* and *R. typhi* co-infection had been found in a surveillance of patients with fever of unknown causes, which were possibly cases of scrub typhus in China (*13*). No co-infection of *O. tsutsugamushi* and *R. typhi* was identified in our study, which might be because relatively fewer cases of murine typhus were identified.

In the 6 patients with tick-borne rickettsioses and *C. burnetii* coinfection reported by Rolain et al., 3 patients likely had concomitant infection caused by tick bites and the other 3 were possibly consecutive infections (*6*). It was difficult to identify concomitant or consecutive infection in our patients because we could not determine a definite time when patients were bitten by arthropod vectors, had contact with animal reservoirs, or were exposed to the environments abundant in *C. burnetii* and *O. tsutsugamushi*. With the assessment of serologic results, however, case-patients 3, 4, and 5 might have acquired scrub typhus first and later been infected with *C. burnetii*; titers of IgM and IgG against *O. tsutsugamushi* were high, and antibodies against *C. burnetii* phase II antigen were negative on first tests in case-patients 4 and 5 (Appendix Table). In case-patient 3 who sought treatment for fever 20 days before admission, the antibody titers for *C. burnetii* phase II antigen and *O. tsutsugamushi* were rising and declining, respectively, in paired serum samples tested. Case-patient 2 might have had Q fever first and scrub typhus later because the first serum tests were positive for *C. burnetii* phase II IgM and IgG, but negative for *O. tsutsugamushi* antibodies. For case-patient 1, the rising antibody titers to both pathogens in paired serum specimens suggest a concomitant infection. Because chiggers were rarely reported as arthropod reservoirs of *C. burnetii*, we believe that our patients might have acquired dual infection through different routes.

The possibility of serologic cross-reaction between Q fever and scrub typhus that results in misdiagnosis of co-infection is low. Cross-reactivity of serologic test results for *C. burnetii* and *Bartonella* spp., *Legionella pneumophila*, *L. micdadei*, and *Ehrlichia chaffeensis* have been reported, but there were no data available on cross-reactivity for *O. tsutsugamushi* (*6**,**14*). In conclusion, although major arthropod vectors, animal reservoirs, and routes of transmission to humans are different for *C. burnetii* and *O. tsutsugamushi*, co-infection with these 2 organisms may occur, particularly in regions where both Q fever and scrub typhus are endemic.

## Supplementary Material

Appendix Table Clinical characteristics and results of examinations of 5 patients with acute Q fever and scrub typhus, Taiwan*

## References

[1] Maurin M, Raoult D. Q fever. Clin Microbiol Rev. 1999;12:518–53. 10515901 10.1128/cmr.12.4.518 PMC88923

[2] Jeong YJ, Kim S, Wook YD, Lee JW, Kim KI, Lee SH. Scrub typhus: clinical, pathologic, and imaging findings. Radiographics. 2007;27:161–72. 10.1148/rg.271065074 17235005

[3] Lai CH, Huang CK, Chin C, Chung HC, Huang WS, Lin CW, Acute Q fever: an emerging and endemic disease in southern Taiwan. Scand J Infect Dis. 2008;40:105–10. 10.1080/00365540701558722 17852909

[4] Lai CH, Huang CK, Weng HC, Chung HC, Liang SH, Lin JN, Clinical characteristics of acute Q fever, scrub typhus, and murine typhus with delayed defervescence despite doxycycline treatment. Am J Trop Med Hyg. 2008;79:441–6. 18784240

[5] Lee HC, Ko WC, Lee HL, Chen HY. Clinical manifestations and complications of rickettsiosis in southern Taiwan. J Formos Med Assoc. 2002;101:385–92. 12189643

[6] Rolain JM, Gouriet F, Brouqui P, Larrey D, Janbon F, Vene S, Concomitant or consecutive infection with *Coxiella burnetii* and tickborne diseases. Clin Infect Dis. 2005;40:82–8. 10.1086/426440 15614696

[7] Janbon F, Raoult D, Reynes J, Bertrand A. Concomitant human infection due to *Rickettsia conorii* and *Coxiella burnetii.* J Infect Dis. 1989;160:354–5. 2760493 10.1093/infdis/160.2.354

[8] Watt G, Jongsakul K, Suttinont C. Possible scrub typhus coinfections in Thai agricultural workers hospitalized with leptospirosis. Am J Trop Med Hyg. 2003;68:89–91. 12556154

[9] Chen YS, Cheng SL, Wang HC, Yang PC. Successful treatment of pulmonary hemorrhage associated with leptospirosis and scrub typhus co-infection by early plasma exchange. J Formos Med Assoc. 2007;106:S1–6. 10.1016/S0929-6646(09)60344-2 17493889

[10] Lu PL, Tseng SH. Fatal septicemic melioidosis in a young military person possibly co-infected with *Leptospira interrogans* and *Orientia tsutsugamushi.* Kaohsiung J Med Sci. 2005;21:173–8. 10.1016/S1607-551X(09)70297-9 15909673 PMC7128880

[11] Wang NC, Ni YH, Peng MY, Chang FY. Acute acalculous cholecystitis and pancreatitis in a patient with concomitant leptospirosis and scrub typhus. J Microbiol Immunol Infect. 2003;36:285–7. 14723261

[12] Lee CH, Liu JW. Co-infection with leptospirosis and scrub typhus in Taiwanese patients. Am J Trop Med Hyg. 2007;77:525–7. 17827372

[13] Zhang LJ, Li XM, Zhang DR, Zhang JS, Di Y, Luan MC, Molecular epidemic survey on co-prevalence of scrub typhus and murine typhus in Yuxi City, Yunnan Province of China. Chin Med J (Engl). 2007;120:1314–8. 17711736

[14] Fournier PE, Marrie TJ, Raoult D. Diagnosis of Q fever. J Clin Microbiol. 1998;36:1823–34. 9650920 10.1128/jcm.36.7.1823-1834.1998 PMC104936

