# Supplementary material for: Acute Q Fever and Scrub Typhus, Southern Taiwan
Source: Emerg Infect Dis. 2009 Oct;15(10):1659–61. doi: 10.3201/eid1510.090007 (PMC2866387; doi:10.3201/eid1510.090007)
Supplement: Appendix Table — Clinical characteristics and results of examinations of 5 patients with acute Q fever and scrub typhus, Taiwan* [file 09-0007_appT-s1.pdf]

Appendix Table. Clinical characteristics and results of examinations of 5 patients with acute Q fever and scrub typhus, Taiwan*

Characteristic	Case-patient no.				
	1	2	3	4	5
Age, y/sex	57/M	21/M	37/M	36/M	51/M
Underlying diseases	DM, hypertension, hepatitis C virus infection	None	Alcoholism, hepatitis B virus infection	None	Hepatitis B virus infection, splenectomy
Clinical symptoms†	Fever, chills, headache, acute cholecystitis, jaundice, skin rash, relative bradycardia	Fever, headache, myalgia	Fever, chills, headache, jaundice, cough, myalgia,	Fever, chills, headache, cough, relative bradycardia	Fever, chills, headache, relative bradycardia
Environmental or animal exposure	Fruit farmer	None	Worker in mountainous area, dogs and chickens at home	School worker in mountainous area	Rice farmer in mountainous area
Laboratory examinations‡	Elevated liver enzyme levels, thrombocytopenia	Elevated liver enzyme levels, thrombocytopenia	Elevated liver enzyme levels, thrombocytopenia	Elevated liver enzyme levels	Elevated liver enzyme levels, thrombocytopenia
Chest radiograph findings	Unilateral infiltration	Normal	Bilateral lower lung infiltration	Normal	Bilateral lower lung infiltration
Abdominal ultrasonography	Gallstone with acute cholecystitis, splenomegaly	NA	Liver cirrhosis, thickened GB wall, splenomegaly, fatty liver	Fatty liver, bilateral renal stones	Thickened GB wall, fatty liver
Q fever testing					
First test, d from disease onset	6	7	21	10	5
Phase 1 IgM/IgG titer	Neg/neg	Neg/neg	≥2,560/320	Neg/neg	Neg/neg
Phase II IgM/IgG titer	Neg/neg	80/320	≥2,560/80	Neg/neg	Neg/neg
Second test, d from disease onset	18	17	28	21	19
Phase I IgM/IgG titer	320/160	160/80	640/320	≥2,560/80	160/320
Phase II IgM/IgG titer	≥2,560/≥2,560	160/≥1,280	≥2,560/≥640	≥2,560/≥2,560	320/≥1,280
Scrub typhus testing					
First test, d from disease onset	6	7	21	10	5

IgM/IgG titer	Neg/neg	Neg/neg	≥320/≥1,280	80/≥1,280	80/≥1,280
Second test, d from disease onset	18	17	28	21	19
IgM/IgG titer	Neg/≥640	80/320	80/320	≥320/≥1,280	≥320/≥1,280
Possible pattern of co-infection	Acute Q fever and scrub typhus concomitantly	Acute Q fever and scrub typhus consecutively	Scrub typhus and acute Q fever consecutively	Scrub typhus and acute Q fever consecutively	Scrub typhus and acute Q fever consecutively
Antimicrobial drug therapy	Cefazolin, gentamicin, metronidazole, doxycycline	Doxycycline	Doxycycline	Doxycycline	Cefazolin, gentamicin, doxycycline

*All 5 patients survived. DM, diabetes mellitus; NA, not available; GB, gallbladder; Ig, immunoglobulin; neg, negative.

†Relative bradycardia, body temperature ≥38.9°C and heart rate <110/min without calcium blockers, beta-blockers, or antiarrhythmic agents.

‡Elevated liver enzyme levels, alanine aminotransferase >44 U/L or aspartate aminotransferase >38 U/L; thrombocytopenia, platelet count <150,000/mm^3.
